# CRISPR/Cas9-mediated mutation of *OsSWEET14* in rice cv. Zhonghua11 confers resistance to *Xanthomonas oryzae* pv. *oryzae* without yield penalty

**DOI:** 10.1186/s12870-020-02524-y

**Published:** 2020-07-03

**Authors:** Xuan Zeng, Yufen Luo, Nga Thi Quynh Vu, Shujuan Shen, Kuaifei Xia, Mingyong Zhang

**Affiliations:** 1 Key Laboratory of South China Agricultural Plant Molecular Analysis and Genetic Improvement, Guangdong Provincial Key Laboratory of Applied Botany, South China Botanical Garden, Chinese Academy of Sciences, Guangzhou, 510650 China; 2 University of Chinese Academy of Sciences, Beijing, 100049 China; 3 Center of Economic Botany, Core Botanical Gardens, Chinese Academy of Sciences, Guangzhou, 510650 China

**Keywords:** Bacterial blight, *OsSWEET14*, Zhonghua 11, AXO1947, Enhanced plant height, No yield penalty

## Abstract

**Background:**

Bacterial blight of rice, caused by *Xanthomonas oryzae* pv. *oryzae* (*Xoo*), is a devastating rice disease in Southeast Asia and West Africa. *OsSWEET14*, encoding a sugar transporter, is known to be a major susceptible gene of bacterial blight targeted by four different transcription activator-like (TAL) effectors from either Asian or African *Xoo* strains. However, the *OsSWEET14* single knockout or promoter mutants in the Kitaake background are moderately resistant or even susceptible to African *Xoo* strains. Therefore, in this study, we knocked out *OsSWEET14* in rice cv. Zhonghua 11 background for disease assessment.

**Results:**

In this study, CRISPR/Cas9 was utilized to disrupt the function of *OsSWEET14* by modifying its corresponding coding region in the genome of rice cv. Zhonghua 11 (*CR-S14*). In total, we obtained nine different *OsSWEET14*-mutant alleles. Besides conferring broad-spectrum resistance to Asian *Xoo* strains, tested mutant alleles also showed strong resistance to African *Xoo* strain AXO1947. Moreover, the expression of *OsSWEET14* was detected in vascular tissues, including the stem, leaf sheath, leaf blade and root. The disruption of *OsSWEET14* led to increased plant height without a reduction in yield.

**Conclusions:**

Disruption of *OsSWEET14* in the Zhonghua 11 background is able to confer strong resistance to African *Xoo* strain AXO1947 and Asian *Xoo* strain PXO86. *CR-S14* has normal reproductive growth and enhanced plant height under normal growth conditions. These results imply that *CR-S14* may serve as a better tester line than *sweet14* single-knockout mutant in the Kitaake background for the diagnostic kit for rice blight resistance. The genetic background and increased plant height need to be taken into consideration when utilizing *OsSWEET14* for resistant rice breeding.

## Background

Bacterial blight of rice, caused by *Xanthomonas oryzae* pv. *oryzae* (*Xoo*), is a devastating rice disease in Southeast Asia and West Africa [1, 2]. The pathogen contains type III effectors that can be injected into rice cells directly via the type III secretion system [3]. Transcription activator-like (TAL) effectors, which are the major virulent effectors in *Xoo*, function like eukaryotic transcription factors to induce target gene expression via binding to the effector-binding elements (EBEs) in the promoter of the target genes [3, 4]. TAL effectors are composed of an N-terminal type III secretion signal, a C-terminal nuclear-localization signal and activation domain and a central repeat domain. The central repeat region consists of 1.5–33.5 tandem repeats that are typically 33–35 amino acids long, and amino acids at the 12th and 13th positions in each repeat are called repeat variable diresidues (RVDs) [5, 6]. The number and order of the RVDs determine the recognition specificity of TAL effectors [5, 6].

*OsSWEETs*, which encode a family of sugar transporters, are classified into three clades phylogenetically [7]. *OsSWEETs* of clade III (*OsSWEET11*–*15*) are reported to be able to induce a susceptible response when induced by artificial TAL effectors [7]. Currently, however, only three of them (*OsSWEET11*, *13* and *14*) are known to be induced by *Xoo* isolated from fields; the corresponding *Xoo* strains that are able to induce the two other *OsSWEET*s (*OsSWEET12* or *OsSWEET15*) have not yet been identified [8–11]. Moreover, *OsSWEET11*, *13* and *14* are the major susceptible targets of *Xoo* [8–10]. *OsSWEET11* is targeted by TAL effector PthXo1 and *OsSWEET13* by PthXo2 or PthXo2-like TAL effectors, while *OsSWEET14* is targeted by four different TAL effectors, i.e., AvrXa7, PthXo3, TalC or Tal5 [7, 8, 10–12]. AvrXa7, PthXo3, PthXo1, PthXo2 and PthXo2-like TAL effectors are present in Asian strains [13]. TalC and Tal5 have only been isolated from African strains, and TalC exists in all the African *Xoo* strains sequenced, while Tal5 is present in half of the strains [13]. Therefore, *OsSWEET14* is the target of all sequenced African *Xoo* strains and most Asian *Xoo* strains.

Since *Xoo* activates *OsSWEET14* by binding to the specific EBEs in the promoter region, great efforts were invested into generating resistant rice plants by genetic editing of the promoter region of *OsSWEET14* or by identifying natural EBE-mutant alleles in germplasm reservoir for resistant rice breeding [14, 15]. The recessive resistance (*R*) gene *xa41(t)*, which is the natural EBE-mutational allele of *OsSWEET14*, has been identified in African rice varieties [16]. *xa41(t)* has an 18 bp deletion in the promoter region overlapping with AvrXa7, PthXo3 and Tal5 EBEs, so it confers resistance to *Xoo* depending on AvrXa7 and PthXo3 for virulence [16]. Since the TalC binding site in the promoter region of *xa41(t)* is intact, and all the sequenced African *Xoo* strains harbor the TalC effector, *xa41(t)* is unable to confer resistance to African *Xoo* strains [13, 16]. Genetically modified rice plants with altered EBEs in the promoter region of *OsSWEET14* showed resistance to *Xoo* depending on the corresponding TAL effector and had normal development [15, 17]. However, a previous study found that mutations in the TalC EBE in the promoter of *OsSWEET14* resulted in a susceptible response to African strain BAI3 that depended on TalC for virulence in the Kitaake background [17]. Recently, researchers found that a mutation in the TalC EBE alone in the Kitaake background still could not confer resistance to African strains; instead a quintuple-mutant promoter lines (rice with mutated PthXo1, PthXo2, TalC, AvrXa7 and Tal5 EBEs) in the Kitaake background were moderately resistant to African strain AXO1947 [13]. Moreover, the *sweet14* single-knockout mutant in the Kitaake background was also susceptible to African strain AXO1947, with a median lesion length of approximately 10 cm, whereas the *sweet13;sweet14* double-knockout mutant showed complete resistance to African strains [18]. All of these previous studies demonstrated that single knockout of *OsSWEET14* in the Kitaake background was unable to confer resistance to African strains. However, *OsSWEET11* and *OsSWEET13* are not target genes of African *Xoo* strains. Theoretically, the mutation of those two genes should not contribute to the resistance response of Kitaake to African *Xoo* strains. The disease response of different mutants cannot be explained with current knowledge.

*OsSWEET14*, encoding a sugar transporter with seven transmembrane helices, targets the plasma membrane and is mainly responsible for sucrose and glucose transportation [19, 20]. TAL effectors function in diverting the nutritional resources from rice by inducing the expression of *OsSWEET14* [19–24]. The *SWEET11* and *SWEET12* single mutant of *Arabidopsis* did not show obvious morphological defects [20]. However, the double mutant was smaller and had an impaired ability to export sucrose from the leaves [20]. Although the rice *OsSWEET14* T-DNA insertion mutant showed AvrXa7- and PthXo3-specific recessive resistance, the homozygous mutant had dramatic development defects including small seeds and severe growth retardation [9]. The homozygous plants required ~ 30 more days to reach the size of 14-day-old normal plants [9].

In our study, we used CRISPR/Cas9 to mutate the coding region of *OsSWEET14* in rice cv. Zhonghua 11 (*CR-S14*) in order to test whether the disruption of *OsSWEET14* in the Zhonghua 11 background will result in broad-spectrum resistance to *Xoo* strains including those originate from Africa. This will help us to know whether *CR-S14* is a better tester line than *sweet14* single-knockout mutant in the Kitaake background for the diagnostic kit for rice blight resistance and also whether the genetic background needs to be taken into consideration when utilizing *OsSWEET14* for resistant rice breeding. Moreover, the assessment of *CR-S14* agronomic traits will help us understand the role of *OsSWEET14* in development and will provide more information when utilizing the *OsSWEET14* knockout mutant for resistant rice breeding.

## Results

### Generation of rice lines edited in the *OsSWEET14* coding region (*CR-S14*)

**Fig. 1:**
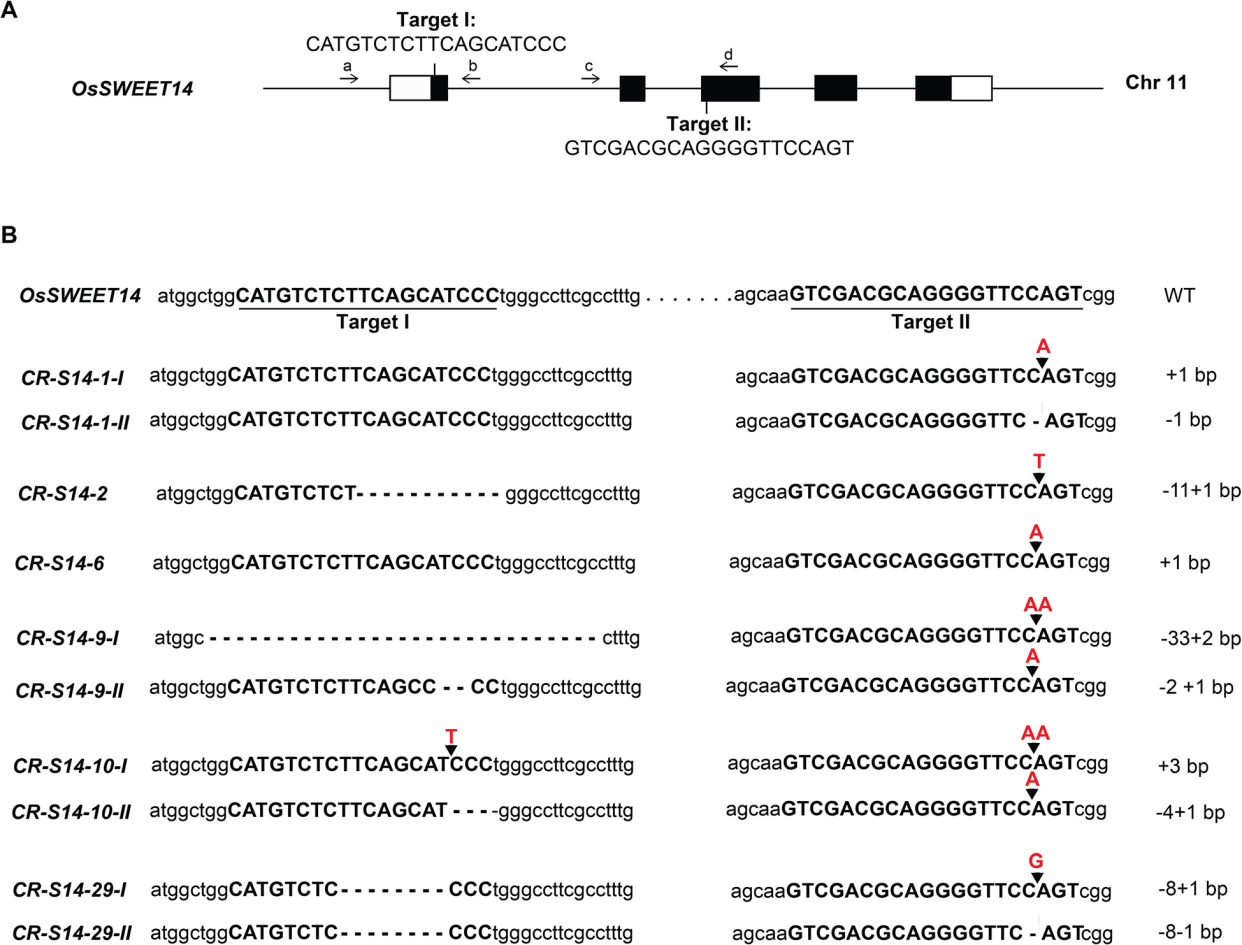
Genetic modification of *OsSWEET14* using CRISPR/Cas9. **a** Two targets were selected in the coding region of *OsSWEET14*. Target I is located in the 1st exon, while target II is located in the 3rd exon. Two pairs of primers (a and b, c and d) were used to amplify the two target regions for sequencing. Black boxes and white boxes represent the corresponding CDS and untranslated region of the *OsSWEET14* transcript in the genome, respectively. a, Target-TalC-F; b, Target-TalC-R; c, Target-S14E3-F; and d, Target-S14E3-R. **b** Sequences of *OsSWEET14* mutant alleles in the target regions. Mutations in *CR-S14–2* and *CR-S14–6* were homozygous in the T_0_ generation, while mutations in *CR-S14–1, CR-S14–9, CR-S14–10* and *CR-S14–29* were biallelic in the T_0_ generation. All the biallelic mutations were homozygous in the T_1_ or T_2_ generation. *CR-S14–1-I* was the same as *CR-S14–6*. Deletions and insertions are indicated by dashes and red letters, respectively. Target I and Target II are underlined in the wildtype (WT). The number of nucleotides that were deleted or inserted in mutant alleles is indicated on the right side of the mutant allele sequence

To modify *OsSWEET14* in rice cv. Zhonghua 11, a CRISPR/Cas9 construct targeting two targets in the corresponding coding region of the *OsSWEET14* genome sequence was built and transformed into Zhonghua 11 background. Target I and Target II were located in the 1st and 3rd exon, respectively (Fig. 1a). Polymerase chain reaction (PCR) and sequencing were used to detect the modifications in the rice transformants. Two rice lines, *CR-S14–2* and *CR-S14–6*, harbored homozygous mutant alleles in the T_0_ generation (Fig. 1b), and the other four lines contained biallelic mutant alleles in the T_0_ generation, i.e., *CR-S14–1*, *CR-S14–9*, *CR-S14–10* and *CR-S14–29*; homozygous mutant alleles were obtained in the T_1_ or T_2_ generation. According to the sequencing result, the modification in mutant allele *CR-S14–1-I* was the same as that in *CR-S14–6*, so a total of nine heritable mutant alleles were obtained (Fig. 1b).

Six of the mutant alleles contained frameshift mutations, i.e., *CR-S14–1-II*, *CR-S14–2*, *CR-S14–6*, *CR-S14–9-I*, *CR-S14–9-II* and *CR-S14–29-I*. The three other mutant alleles, *CR-S14–10-I*, *CR-S14–10-II* and *CR-S14–29-II*, contained in-frame mutations (Fig. 1b). Mutations were also amplified and confirmed in several mutant transcripts (Additional file 1). Since OsSWEET14 is a sugar transporter containing seven transmembrane helices, the function of the protein depends strongly on the helices. The TMHMM2.0 program was utilized to predict the transmembrane helices of proteins encoded by mutant alleles [25]. All of the frameshift mutant alleles encoded proteins with only one or even no transmembrane helices, while all the in-frame mutant alleles encoded proteins containing five or six transmembrane helices (Additional files 2 and 3). Loss of the transmembrane helices had high possibilities of disrupting the transporter activity. This may indicate that all the *OsSWEET14* mutant alleles encode proteins without sugar transportation ability.

### *CR-S14* conferred strong resistance to African *Xoo* strain AXO1947

Zhonghua 11 contained the recessive resistance allele of *OsSWEET13*, which was a deletion in the PthXo2 EBE region leading to the incapability of being recognized by PthXo2 (Additional file 4). PXO86 is a Philippines-originated *Xoo* that depended on AvrXa7 to activate *OsSWEET14* expression for virulence, while T7174 (NCBI: txid342109) is a Japanese strain harboring both AvrXa7 and PthXo2 for virulence. Consistent with *OsSWEET14* knockout mutant in the Kitaake background reported previously, *CR-S14* conferred strong resistance to both PXO86 and T7174 (Additional file 5 and Table 1) [18]. In addition, *CR-S14* showed a broad resistance to the tested Asian *Xoo* strains (Table 1).

**Table 1:** Disease resistance evaluation of *Xoo* strains from different regions. At least 10 leaves of three plants were inoculated for each *Xoo* strain

Strain^a^	Strain origin	Lesion length and disease response^b^	
		Zhonghua 11	*CR S14–6*^c^
GD1358	China	8.11 ± 3.86 (MS)	0.26 ± 0.29 (R)
JS49–6	China	11.9 ± 2.50 (S)	0.87 ± 0.72 (R)
HB17	China	19.5 ± 8.23 (S)	1.74 ± 16.1 (R)
HB21	China	15.05 ± 5.85 (S)	0.14 ± 0.06 (R)
HLJ72	China	12.91 ± 4.87 (S)	0.65 ± 0.72 (R)
NX42	China	13.00 ± 7.24 (S)	0.63 ± 0.88 (R)
HN1–2	China	16 ± 5.62 (S)	0.7 ± 1.12 (R)
LC-4	China	17.05 ± 7.26 (S)	1.04 ± 1.25 (R)
IV-1	China	14.64 ± 5.36 (S)	0.43 ± 0.43 (R)
PXO79	Philippines	7.26 ± 2.86 (MS)	0.49 ± 0.42 (R)
PXO86	Philippines	8.17 ± 3.05 (MS)	0.25 ± 0.31 (R)
PXO71	Philippines	8.59 ± 2.94 (MS)	10.27 ± 2.44 (S)
Aust2031	Australia	8.78 ± 5.53 (MS)	0.24 ± 0.25 (R)
T7174	Japan	8.27 ± 8.44 (MS)	0.35 ± 0.28 (R)
A3857	India	10.57 ± 3.21 (S)	10.19 ± 3.61 (S)
A3842	India	10.11 ± 2.47 (S)	11.66 ± 2.48 (S)

^a^*Xoo* strains were cultivated two days on PSA medium and inoculated on six-week-old rice plants

^b^ Lesions length was scored 14 days after inoculation. R, resistant, lesion length < 3.0 cm; MS, moderately susceptible, 6.0 cm < lesion lenght≤9.0 cm; S, susceptible, lesion length > 9.0 cm

^c^*CR S14–6*, one of the homozygous *OsSWEET14* knockout mutant in Zhonghua 11 background

**Fig. 2:**
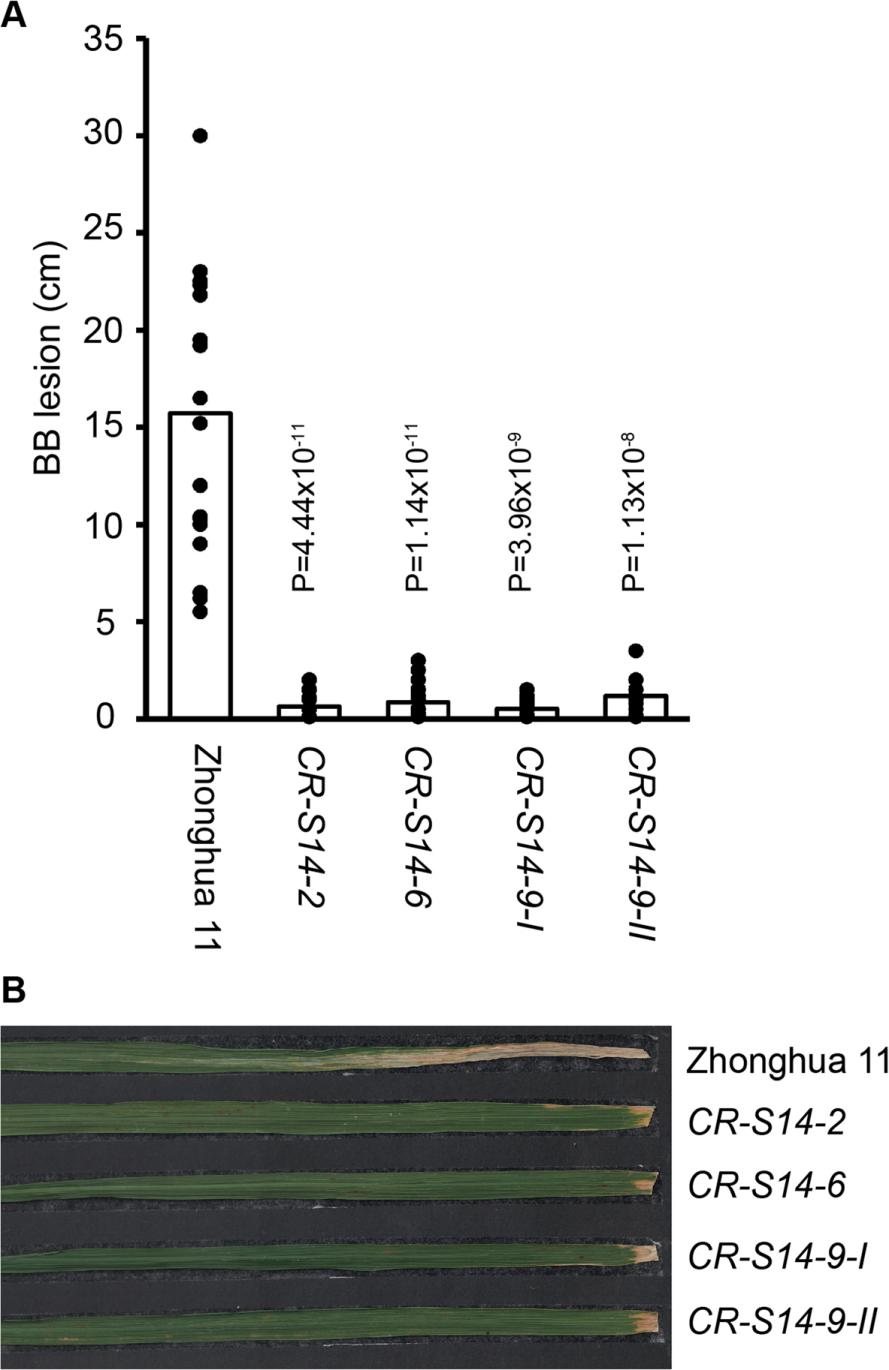
*CR-S14* confers strong resistance to AXO1947. **a** Lesion lengths of *CR-S14* and Zhonghua 11 inoculated with AXO1947 that harbored TalC at 14 days after inoculation (*n* > 15). **b** Phenotype of *CR-S14* and Zhonghua 11 at 14 days after inoculation with AXO1947. *CR-S14–2*, *CR-S14–6*, *CR-S14–9-I* and *CR-S14–9-II* rice plants harboring different homozygous *OsSWEET14* mutant alleles in the Zhonghua 11 background. Zhonghua 11, wildtype control. Six-week-old rice plants were inoculated with AXO1947. Lesion lengths on inoculated leaves were scored 14 days after inoculation. Statistical analysis was performed using a two-tailed Student’s *t* test against Zhonghua 11

To test the disease response of *CR-S14* to *Xoo* strains that depended on TalC for virulence, an Africa-originated *Xoo* strain (AXO1947) harboring TalC was inoculated on *CR-S14* and Zhonghua 11 by the leaf clipping method. At least three plants of each mutant allele were inoculated with AXO1947. Fourteen days after inoculation, Zhonghua 11 showed a susceptible response to AXO1947, while *CR-S14* conferred strong resistance to AXO1947 with an average lesion length less than 2 cm (Fig. 2). This demonstrated that the disruption of *OsSWEET14* in the Zhonghua 11 background conferred strong resistance to AXO1947.

To determine whether the mutant alleles were still inducible, PXO86 and sterile H_2_O were inoculated on *CR-S14* and Zhonghua 11 with syringes. The expression level of *OsSWEET14* and the mutant alleles were measured at 0 and 48 h after inoculation using qRT-PCR. PXO86 was able to induce the expression of both mutant alleles in *CR-S14* and *OsSWEET14* in Zhonghua 11 (Additional file 5). This result demonstrated the mutant alleles were still inducible by AvrXa7, and the resistance response was caused by the disruption of OsSWEET14 transporter activity.

### *CR-S14* had enhanced plant height and normal reproductive growth

**Fig. 3:**
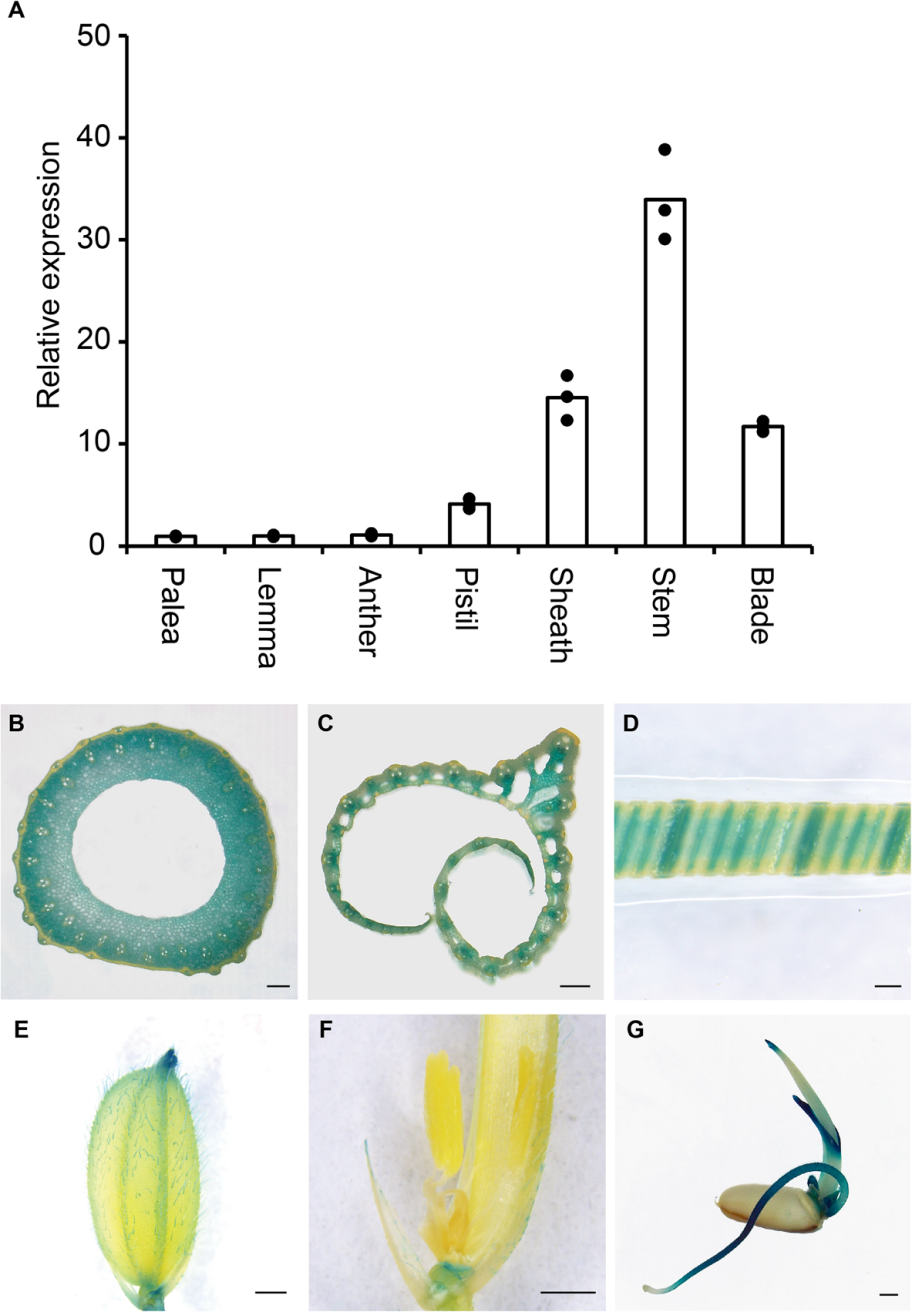
Tissue-specific expression of *OsSWEET14*. **a** Detection of *OsSWEET14* transcripts in different rice tissues by qRT-PCR. **b**–**g** GUS staining in different tissues of *pOsSWEET14:GUS* transgenic plants in the Zhonghua 11 background, including the stem (**b**), leaf sheath (**c**), leaf blade (**d**), spikelet (**e**), anther (**f**) and seedling (**g**). Scale bars, 250 μM (**b**–**d**) and 1 mm (**e**–**g**)

*OsSWEET14* transcripts were detected in various tissues of rice plants using qRT-PCR; the highest levels were measured in the stem, followed by leaf sheaths and blades (Fig. 3a). The expression pattern of *OsSWEET14* was further investigated in *pOsSWEET14:GUS* transgenic rice plants using a β-glucuronidase (GUS) reporter gene under the control of the *OsSWEET14* promoter. Consistent with the qRT-PCR result, strong GUS activity was detected in most of the cell types in the stem and mainly in the veins of leaf sheath and blades (Fig. 3b and d). Consistent with the expression pattern of *OsSWEET14* in the RiceXPro database [26], GUS activity was also highly detected in the roots; however, root tips, which do not have vascular bundles, lacked GUS activity (Fig. 3g). Low GUS activity was detected in the spikelet, including the palea, lemma and anther (Fig. 3e and f). This indicated that *OsSWEET14* is mainly expressed in the vascular tissues of rice plants.

**Fig. 4:**
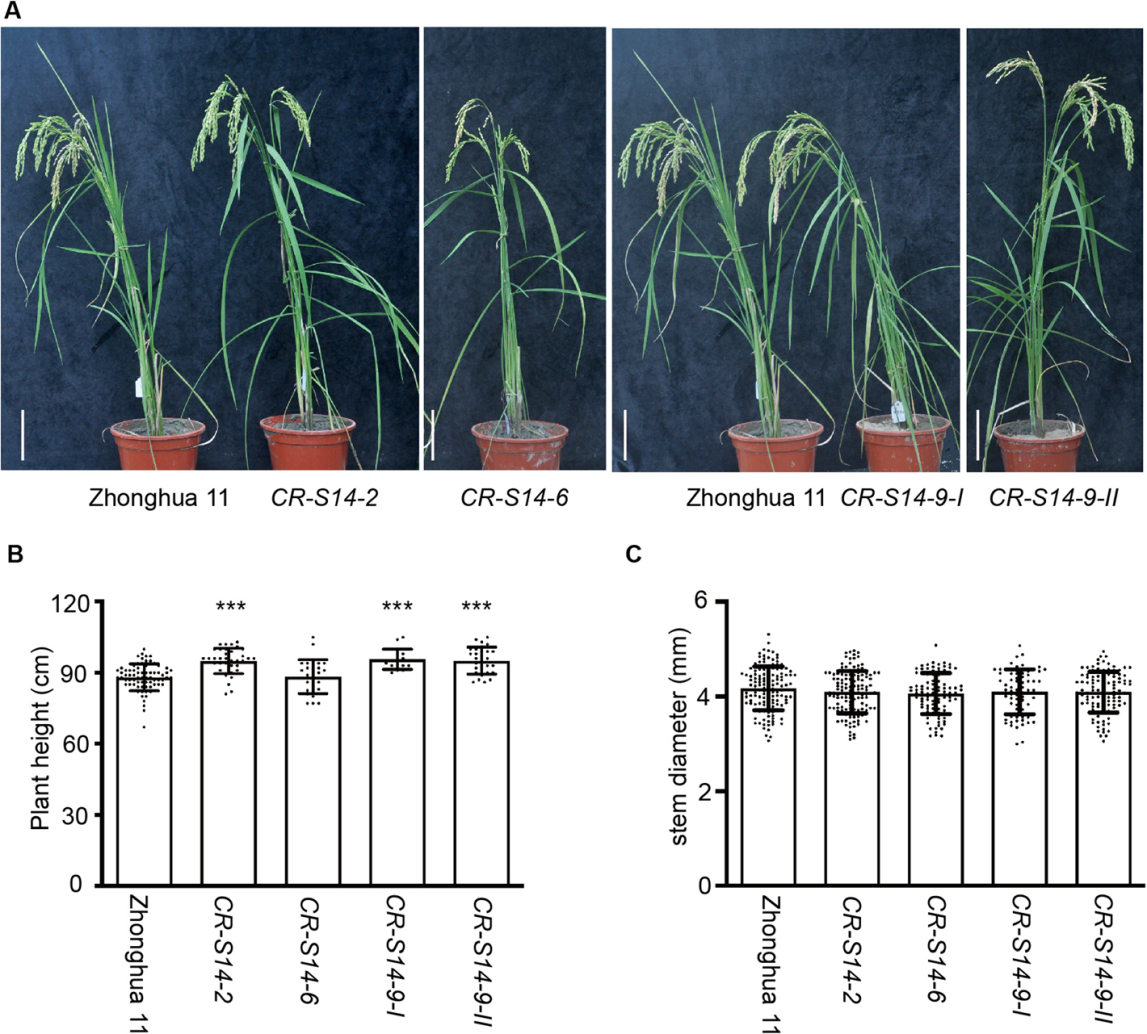
*CR-S14* had enhanced plant height. **a***CR-S14* did not show obvious morphological defects under normal growth conditions at the mature stage. **b**, **c** Performance of *CR-S14* plants in terms of height (**b**) and stem diameter (**c**) relative to Zhonghua 11 plants at the mature stage. *CR-S14–2*, *CR-S14–9-I* and *CR-S14–9-II* were significantly taller than Zhonghua 11 (*P* < 0.001). The plant height of *CR-S14–6* was comparable to that of Zhonghua 11 (*P* > 0.05). No significant difference was detected in the stem diameter between mutant lines and Zhonghua 11 (*P* > 0.05). Scale bar, 10 cm. Statistical analysis was performed using a two-tailed Student’s *t* test against Zhonghua 11, and the significance is indicated by asterisks as follows: 0.01 < *P* < 0.05 (*), 0.001 < *P* < 0.01 (**), *P* < 0.001 (***). *n* > 15

To verify the influence of *OsSWEET14* disruption on rice development, *CR-S14* and Zhonghua11 were grown in a paddy field in Guangdong Province in China. *CR-S14* and Zhonghua 11 were germinated and transferred to the paddy field at the same time, and no growth retardation was observed (Fig. 4a). Since *OsSWEET14* transcripts accumulated highly in the stem, we tested whether the knockout of *OsSWEET14* affected the stem diameter and plant height at the mature stage. Statistical analysis was performed using a two-tailed Student’s *t* test against Zhonghua 11. No significant difference (*P* > 0.05) in stem diameter was detected between *CR-S14* and Zhonghua 11 (Fig. 4c and Additional file 6). However, *CR-S14–2*, *CR-S14–9-I* and *CR-S14–9-II* were approximately 7 cm taller than Zhonghua 11, which equates to an approximate 8% increase in plant height (Fig. 4b and Additional file 6). The stem diameter and plant height were recorded for two seasons in 2019 in Guangdong, China. This suggested that the disruption of *OsSWEET14* led to an increase in plant height.

**Fig. 5:**
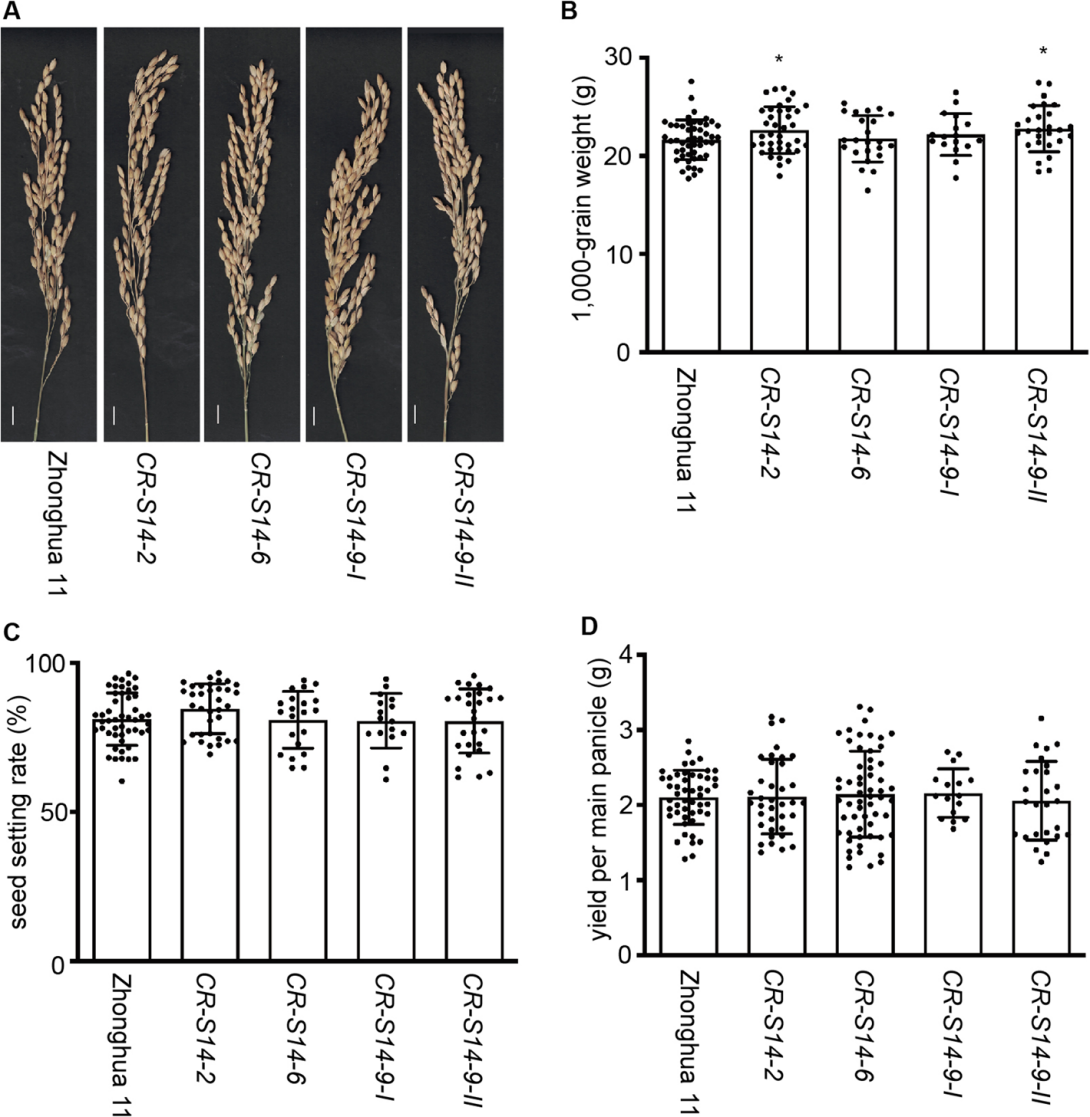
*CR-S14* had normal reproductive growth in the paddy field. **a** A comparison of the main panicles at the mature stage. No obvious defects were observed under normal growth conditions. **b**–**d** 1000-grain weight (**b**), seed setting rate (**c**) and yield (**d**) of the *CR-S14* main panicle relative to that of Zhonghua 11 at the mature stage. The 1000-grain weight of *CR-S14–2* and *CR-S14–9-II* was slightly higher than that of Zhonghua 11 (0.01 < *P* < 0.05). *CR-S14–6* and *CR-S14–9-I* had a similar 1000-grain weight to that of Zhonghua 11. No significant difference between *CR-S14* and Zhonghua 11 was detected in the seed setting rate and yield. Scale bar, 1 cm. Statistical analysis was performed using a two-tailed Student’s *t* test against Zhonghua 11, and the significance is indicated by asterisks as follows: 0.01 < *P* < 0.05 (*), 0.001 < *P* < 0.01 (**), *P* < 0.001 (***). *n* > 15

In addition, we checked whether the disruption of *OsSWEET14* affected the reproductive growth of rice. At least 15 plants of each mutant allele and 30 Zhonghua 11 plants were grown in the paddy field in Guangzhou, Guangdong, China. The 1000-grain weight, seed setting rate and yield of the main panicles were assessed. The assessment was executed for two seasons in 2019. The 1000-grain weight of *CR-S14–2* and *CR-S14–9-II* was slightly higher (*P* < 0.05) than that of Zhonghua 11, while *CR-S14–6* and *CR-S14–9-I* did not differ significantly (*P* > 0.05) from Zhonghua 11 (Fig. 5 and Additional file 6). This suggested that the disruption of *OsSWEET14* did not affect the reproductive growth of rice plants under normal growth conditions. All these results indicated that the disruption of *OsSWEET14* increased plant height without reducing yield under normal growth conditions.

## Discussion

In natural germplasm reservoirs, recessive resistant alleles that harbor disrupted EBEs in the promoter region are able to confer resistance to *Xoo* strains depending on the corresponding TAL effectors for virulence. However, TAL effectors are able to adapt to the recessive resistance alleles under the selection pressure from the resistant varieties. Recently, many PthXo2-like, AvrXa7/PthXo3-like, TalC-like and PthXo1-like TAL effectors have been identified in *Xoo* strains that are able to activate the expression of *OsSWEET13*, *OsSWEET14* or *OsSWEET11* in different rice varieties [12, 13]. For example, *OsSWEET13*_Kit_ in the Kitaake background, which is the natural EBE-mutational allele of *OsSWEET13*, is non-inducible by PthXo2. However, two PthXo2-like TALE effectors (Tal5_LN18_ and Tal7_PXO61_) identified recently are able to activate the expression of *OsSWEET13*_Kit_ to induce susceptible response [12]. These results indicate that recessive resistance can be overcome by the emergence of novel TAL effectors, and the engineering of the promoter region of *OsSWEET*s genes is not sufficient to confer durable and broad-spectrum resistance to *Xoo*. As a result, we directly knocked out *OsSWEET14* in the Zhonghua 11 background directly (*CR-S14*) in order to confer resistance to all the *Xoo* strains that depend on *OsSWEET14* induction for virulence since Zhonghua 11 contains the recessive PthXo2 EBE mutant allele (Additional file 4). *CR-S14* was able to confer complete resistance to strains depending on both PthXo2 and AvrXa7 for virulence, such as *Xoo* strain T7174 (Table 1). Furthermore, *CR-S14* also conferred complete resistance to African strain AXO1947, which depends on TalC for *OsSWEET14* induction. However, the disease response was inconsistent with that in the Kitaake background. *OsSWEET14* knockout mutants or *OsSWEET14* promoter mutants in the Kitaake background are susceptible to AXO1947 [13, 18]. This inconsistence implies other susceptible targets could be induced in the Kitaake genetic background instead of Zhonghua 11. In 2018, one research paper reported that TAL effector TalB_MAI1_ from the African *Xoo* strain MAI1 was able to activate the expression of two susceptible genes (*OsTFX1* and *OsERF#123*) in the Nipponbare background and induce a susceptible response [27]. This suggested that African *Xoo* strains might be able to induce other susceptible genes in the Kitaake background besides *OsSWEET14* for virulence. The *OsSWEET14* single knockout mutant in the Kitaake background was susceptible to AXO1947. This indicated that the genetic background of rice varieties may affect the resistance response of the *OsSWEET14* knockout mutant. This hypothesis can be verified by hybridizing *CR-S14* with Kitaake and checking the resistance response of F_2_ generation plants. If novel susceptible targets exist in the Kitaake background, F_2_ generation plants harboring the homozygous *CR-S14* allele will show disease-response phenotype segregation. Another possibility causing this disease response difference is the off-target effect. Mutagenesis of *OsSWEET14* in the Zhonghua 11 background was mediated by CRISPR/Cas9 targeting of two target sites, Target I was in the 1st exon, and Target II was in the 3rd exon (Fig. 1). Mutagenesis of *OsSWEET14* in the Kitaake background was mediated by only one target site that is the same site as Target I in Zhonghua 11 (Kittake target: 5′-GCATGTCTCTTCAGCATCCCTGG-3′ vs. Target I (*CR-S14*): 5′-CATGTCTCTTCAGCATCCCTGG-3′). This implies that the off-target effect of Target II in Zhonghua 11 might lead to a difference in the disease response. This can be verified by generating a new *OsSWEET14* knockout mutant in Zhonghua 11 background with CRISPR/Cas9 targeting to Target I or other different target sites.

TalC and Tal5 are two TAL effectors that target different EBEs in the promoter region of *OsSWEET14*. All the sequenced African *Xoo* strains habor TalC or both TalC and Tal5, which indicates that all the African *Xoo* strains are able to activate *OsSWEET14* [13]. The *OsSWEET14* knockout mutant in the Kitaake background is susceptible to Africa-originated *Xoo* strains, while *CR-S14* is able to confer strong resistance to AXO1947. This implies *CR-S14* may serve as a better tester line than *sweet14* single-knockout mutant in the Kitaake background for the diagnostic kit for rice blight resistance [18].

*CR-S14* plants were taller than Zhonghua 11 and had no reproductive defects under normal growth condition. Since *OsSWEET14* had the highest expression level in the stem, the enhanced plant height might be due to the lower efficiency of sugar transportation in the stem, which needs to be studied further. In addition, *OsSWEET14* may not be responsible for reproductive development since its expression in the anther, palea, lemma and pistil was relatively low. These results imply the enhancement of plant height needs to be taken into consideration if knockout mutants of *OsSWEET14* are used to confer resistance to bacterial blight. The *sweet14* single-knockout mutant in the Kitaake background also showed normal growth as reported [18]. However, the homozygous *OsSWEET14* T-DNA insertion mutant showed dramatic developmental defects [9]. This might be due to multiple T-DNA insertions in the rice genome, which were not discussed in paper [9].

Through comparison with the 3,010 rice genomes from 3,000 Rice Genome Project [28], we found most SNPs or indels in the *OsSWEET14* genome region were in untranslated regions or introns. Two and four SNPs were identified in the 3rd and 5th exons, respectively, and 23 different indels were found in the 5th exon. However, all the indels are in the C terminal of OsSWEET14 and do not affect the transmembrane helices. Therefore, whether those indels abrogate the function of OsSWEET14 needs to be tested further. In addition, the natural *OsSWEET14* non-functional alleles need to be discovered.

## Conclusions

This study demonstrated that the disruption of *OsSWEET14* in the Zhonghua 11 background is able to confer strong resistance to African *Xoo* strain AXO1947 and Asian strain PXO86 with enhanced plant height and no yield penalty. These results imply that *CR-S14* might be a better tester line than *sweet14* single-knockout mutant in the Kitaake background for diagnostic kit for rice blight resistance. The different disease responses between *CR-S14* and the *ossweet14* single knockout mutant in the Kitaake background might be due to the genetic background or off-target effect. This hypothesis needs to be experimentally confirmed. Moreover, natural non-functional *OsSWEET14* mutant alleles need to be identified for further rice breeding applications. Finally, the enhancement of plant height needs to be taken into consideration when utilizing *OsSWEET14* for resistant rice breeding.

## Methods

### Rice growth conditions

Zhonghua 11 (*Oryza sativa* L. ssp. *Japonica* cv. Zhonghua 11) was used in this study. *CR-S14* is a genetically modified rice plants in the Zhonghua 11 background. At least 15 plants of different *CR-S14* lines and more than 30 Zhonghua 11 plants were grown in a field under optimum growing conditions. We grew both *CR-S14* and Zhonghua 11 for two seasons in 2019; the first season was from March to June, and the second season was from August to November. The paddy field is located in Guangzhou, Guangdong, China.

### Bacteria strains and inoculation

All *Xoo* strains were cultivated on PSA medium (10 g/l peptone, 10 g/l sucrose, 1 g/l glutamic acid, 16 g/l agar, and pH 7.0) at 28 °C for 2 days. Bacteria were suspended in sterile water to an OD_600_ 0.5 for inoculation on rice leaves. Bacterial blight inoculation was executed with the leaf-clipping method as previously described [29]. Inoculated rice leaves were collected 48 h after inoculation for gene induction analysis. Bacterial lesions were measured 14 days after inoculation. The sources of the *Xoo* strains tested in this study are listed in Additional file 7.

### Gene expression analysis

The total RNA of rice leaves was extracted using an Eastep® Super Total RNA Extraction Kit (Promega) and reverse-transcribed into single-stranded cDNA using GoScript™ Reverse Transcription Mix, Oligo (dT) (Promega, USA). Real-time quantitative RT-PCR was executed on LightCycler480 (Roche, Switzerland) using iTaq Universal SYBR Green Supermix (Bio-rad, USA). The expression of rice ubiquitin gene 5 (*Ubi5*) was used as the internal reference gene. The specific primer pair for *Ubi5* was 5′-AACCACTTCGACCGCCACT-3′ and 5′-GTTCGATTTCCTCCTCCTTCC-3′. All the primers used in this study are listed in Additional file 8.

### Construct and rice transformation

Two target sites were selected on the 1st and 3rd exon of *OsSWEET14* and were inserted into the CRISPR/Cas9 vector as described [30]. A 2-kb promoter fragment upstream of the *OsSWEET14* transcriptional initiation site was amplified and inserted in front of the *GUS* reporter gene in the pCAMBIA1301 vector. The plasmid was transformed into *Agrobacterium* EHA105. Rice transformation of Zhonghua 11 was performed as previously described [31].

### Determination of mutations in *CR-S14*

The regions flanking the two targets on *OsSWEE14* in independent T_0_ generation hygromycin-resistant *CR-S14* lines were amplified with two pairs of primers, Target-TalC-F & R and Target-S14E3-F & R, and were sequenced to determine the potential alterations. For those rice lines with biallelic mutations, modifications were determined in segregating progeny (T_1_) of self-pollinated T_0_ plants. All the primers used in this study are listed in Additional file 8.

### Determination of *CR-S14* morphology

To determine the morphology of *CR-S14*, at least 15 plants of each line were measured. The upper diameter of the second stem segment of every productive tiller was measured as the stem diameter. The length of the mature rice plant from the ground to the tip of the panicle was measured as the plant height. The grain weight, number of filled grains and number of empty grains were weighed and counted with a seed morphology inspection machine (SC-G, wseen, China). Subsequently, the 1000-grain weight, seed setting rate and yield of the main panicle were calculated.

### Histochemical analysis of GUS activity

Rice tissues were cut and transferred into a 2 ml microcentrifuge tube containing GUS staining solution (50 mM sodium phosphate, pH 7.0, 7% methanol, and 1 mM 5-bromo-4-chloro-3-indolyl-β-d-glucuronide) overnight at 37 °C and subsequently destained in an ethanol series. Stained rice tissues were observed under a stereomicroscope.

## Supplementary information

**Additional file 1. **Detection of mutations in *CR-S14* transcripts.

**Additional file 2.** Prediction of transmembrane helices in OsSWEET14 and modified OsSWEET14.

**Additional file 3.** Predicted amino acid sequence of CR-S14.

**Additional file 4. ***OsSWEET13*_ZH11_ EBE sequence.

**Additional file 5. ***CR-S14* confers strong resistance to PXO86.

**Additional file 6. **Agronomic traits of *CR-S14*.

**Additional file 7. **Source of *Xoo* strains.

**Additional file 8.** Primers used in this study.

## Data Availability

The datasets supporting the conclusions of this article are included within the article and its additional files.
