# Supplementary material for: CRISPR/Cas9-mediated mutation of OsSWEET14 in rice cv. Zhonghua11 confers resistance to Xanthomonas oryzae pv. oryzae without yield penalty
Source: BMC Plant Biol. 2020 Jul 3;20:313. doi: 10.1186/s12870-020-02524-y (PMC7333420; doi:10.1186/s12870-020-02524-y)
Supplement: Supplementary file 1 — Additional file 1. Detection of mutations in CR-S14 transcripts. [file 12870_2020_2524_MOESM1_ESM.pdf]

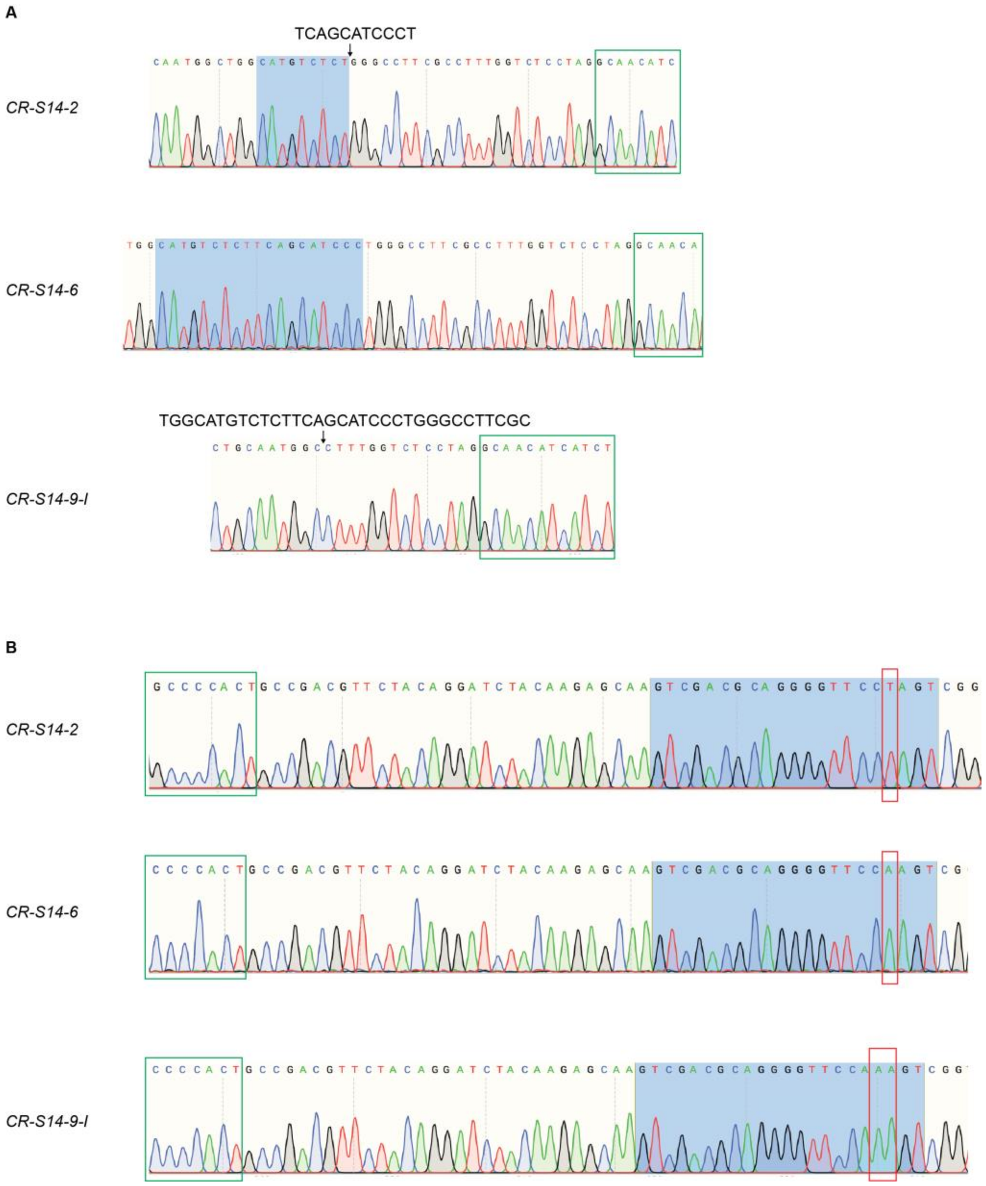

Additional file 1 Detection of mutations in *CR-S14* transcripts. (A) Sanger sequencing chromatograms of *CR-S14-2*, *CR-S14-6* and *CR-S14-9-I* target I regions. (B) Sanger sequencing chromatograms of *CR-S14-2*, *CR-S14-6* and *CR-S14-9-I* target II regions. Total RNAs were extracted from T_2 generation of *CR-S14* mutant lines for cDNA synthesis and PCR amplification. Black arrows indicate the deletion site and deleted nucleotides were shown above the arrow. Red boxes indicate the inserted nucleotides. Green boxes indicate sequence of the 2nd exon. Target sites were indicated with blue background. Chromatograms of sequencing results were visualized with SnapGene Viewer.
