# Supplementary material for: CRISPR/Cas9-mediated mutation of OsSWEET14 in rice cv. Zhonghua11 confers resistance to Xanthomonas oryzae pv. oryzae without yield penalty
Source: BMC Plant Biol. 2020 Jul 3;20:313. doi: 10.1186/s12870-020-02524-y (PMC7333420; doi:10.1186/s12870-020-02524-y)
Supplement: Supplementary file 2 — Additional file 2. Prediction of transmembrane helices in OsSWEET14 and modified OsSWEET14. [file 12870_2020_2524_MOESM2_ESM.pdf]

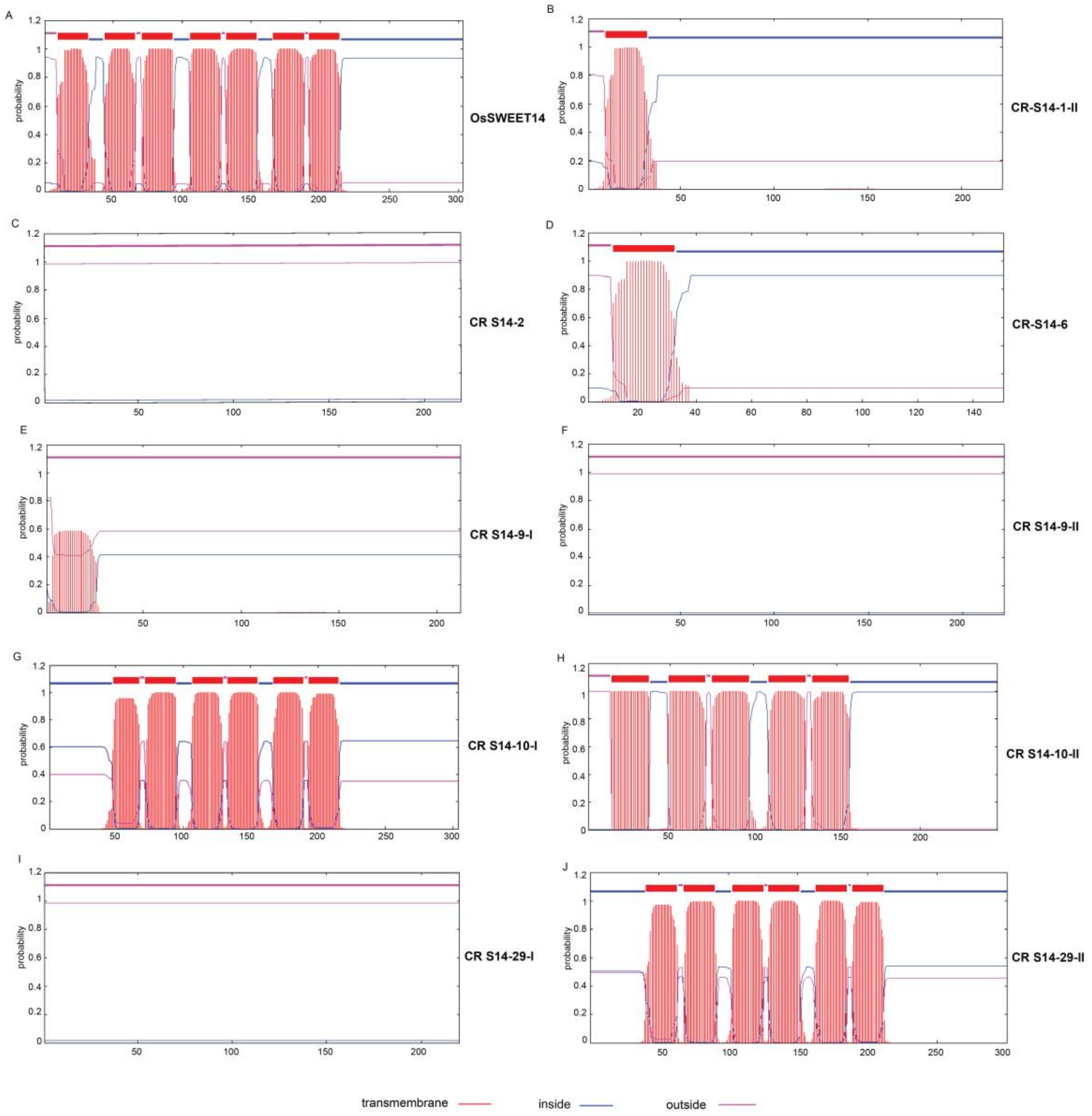

Additional file 2 Prediction of transmembrane helices in OsSWEET14 and modified OsSWEET14. (A) OsSWEET14 contains seven helices, (B) CR-S14-1-II, (C) CR-S14-2, (D) CR-S14-6, (E) CR-S14-9-I, (F) CR-S14-9-II and (I) CR-S14-29-I are frameshift mutants and have no or only one transmembrane helix, (G) CR-S14-10-I, (H) CR-S14-10-II and (J) CR-S14-29-II are in-frame mutant that contain five or six transmembrane helices. The transmembrane helices were predicted by the TMHMM2.0 program (http://www.cbs.dtu.dk/services/TMHMM/).
