# Supplementary material for: CRISPR/Cas9-mediated mutation of OsSWEET14 in rice cv. Zhonghua11 confers resistance to Xanthomonas oryzae pv. oryzae without yield penalty
Source: BMC Plant Biol. 2020 Jul 3;20:313. doi: 10.1186/s12870-020-02524-y (PMC7333420; doi:10.1186/s12870-020-02524-y)
Supplement: Supplementary file 3 — Additional file 3. Predicted amino acid sequence of CR-S14. [file 12870_2020_2524_MOESM3_ESM.pdf]

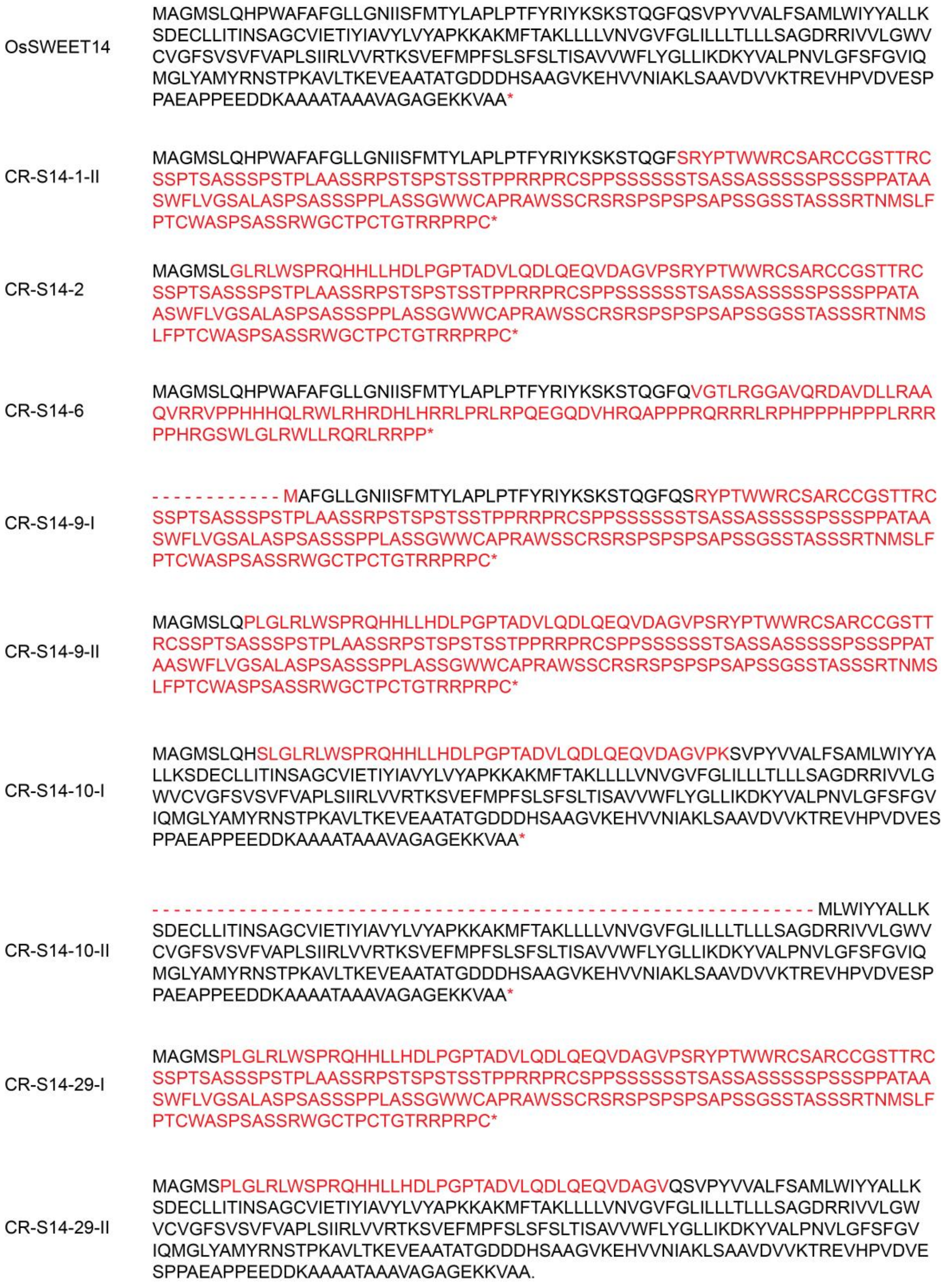

Additional file 3 Predicted amino acid sequence of CR-S14. Frameshifts occur at target I in CR-S14-2, CR-S14-9-II and CR-S14-29-I and occur at target II in CR-S14-1-II, CR-S14-6 and CR-S14-9-I, leading to polypeptides with altered sequence and length. Frameshifts also

occur between target I and target II in CR-S14-10-I and CR-S14-29-II leading to loss of the first transmembrane helix. Frameshifts occur at target I in CR-S14-10-II leading to polypeptides with 18 amino acids due to premature stop codon. However, there is another start codon (*ATG*) 120 bp downstream of the premature stop codon this might lead to the coding of a truncated OsSWEET14 protein without the first two transmembrane helices.
