# Supplementary material for: CRISPR/Cas9-mediated mutation of OsSWEET14 in rice cv. Zhonghua11 confers resistance to Xanthomonas oryzae pv. oryzae without yield penalty
Source: BMC Plant Biol. 2020 Jul 3;20:313. doi: 10.1186/s12870-020-02524-y (PMC7333420; doi:10.1186/s12870-020-02524-y)
Supplement: Supplementary file 4 — Additional file 4. OsSWEET13ZH11 EBE sequence. [file 12870_2020_2524_MOESM4_ESM.pdf]

A

OsSWEET13$_{IR24}$ EBE: TATAAAAGCACCACAACTCCCTT

OsSWEET13$_{ZH11}$ EBE:TATAAA - GCACCACAACTCCCTT

B

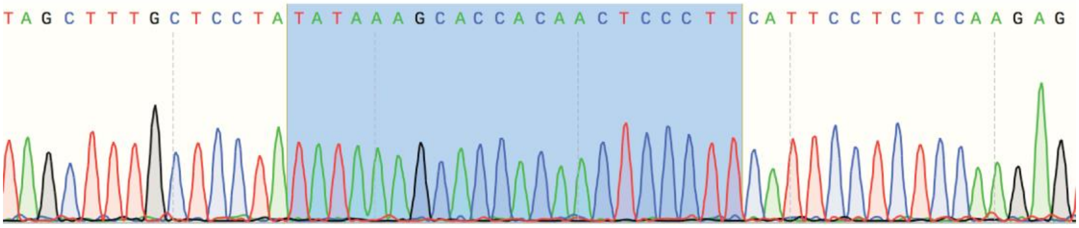

Additional file 4 *OsSWEET13*$_{ZH11}$ EBE sequence. (A) *OsSWEET13*$_{IR24}$ EBE and *OsSWEET13*$_{ZH11}$ EBE sequences. (B) Sanger sequencing chromatograms of *OsSWEET13* promoter region in rice cv. Zhonghua 11. Sequence of *OsSWEET13*$_{ZH11}$ EBE is the same with *OsSWEET13*$_{Kit}$ EBE, which can be recognized by Tal5$_{LN18}$ ad Tal7$_{PXO61}$. *OsSWEET13*$_{IR24}$ EBE can be recognized by TAL effector PthXo2. *OsSWEET13*$_{ZH11}$ EBE were highlighted with blue background.
