# Supplementary material for: CRISPR/Cas9-mediated mutation of OsSWEET14 in rice cv. Zhonghua11 confers resistance to Xanthomonas oryzae pv. oryzae without yield penalty
Source: BMC Plant Biol. 2020 Jul 3;20:313. doi: 10.1186/s12870-020-02524-y (PMC7333420; doi:10.1186/s12870-020-02524-y)
Supplement: Supplementary file 5 — Additional file 5. CR-S14 confers strong resistance to PXO86. [file 12870_2020_2524_MOESM5_ESM.pdf]

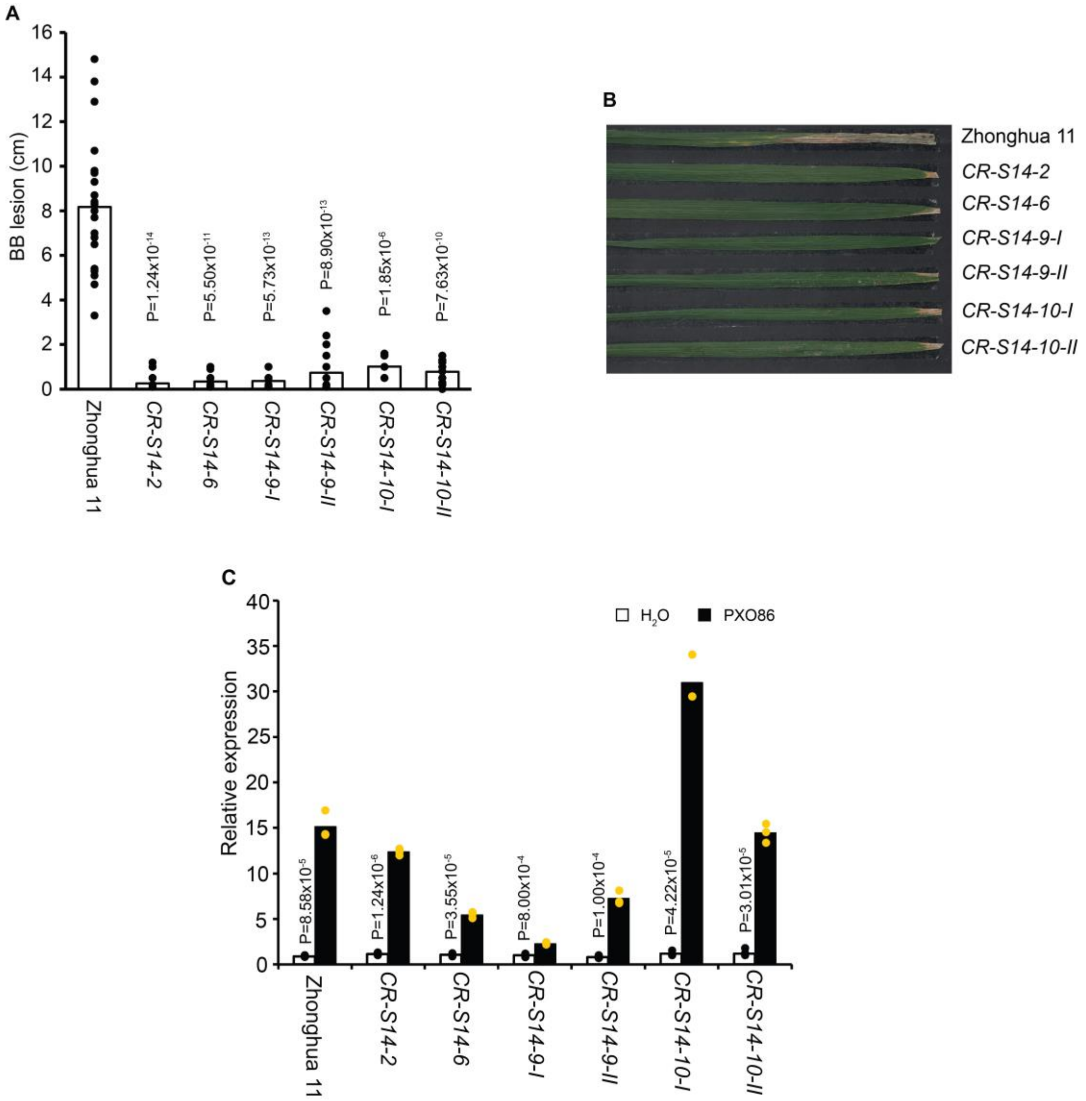

Additional file 5 *CR-S14* confers strong resistance to PXO86. (A) Lesion lengths of *CR-S14* and Zhonghua 11 inoculated with PXO86 which harboring AvrXa7 at 14 days after inoculation (n>15). (B) Phenotype of *CR-S14* and Zhonghua 11 at 14 days after inoculation with PXO86. *CR-S14-2*, *CR-S14-6*, *CR-S14-9-I*, and *CR-S14-9-II* are rice plants harbor different homozygous *OsSWEET14* mutant alleles in Zhonghua 11 background. Lesion lengths on inoculated leaves were scored 14 days after inoculation. (C) Induction of mutant alleles by PXO86. Leaves of six-week-old rice plants were inoculated with PXO86 and H_2O by syringes and collected at 48 hours after inoculation. Zhonghua 11 was used as control. The

expression level of *OsSWEET14* in Zhonghua 11 or *OsSWEET14* mutant alleles in genetic modified plants inoculated with PXO86 was normalized to those inoculated with H_2O which were all set as ‘1’. Rice ubiquitin gene 5 (*Ubi5*) was used as reference gene. Statistical analysis was performed using two-tailed Student’s *t* test.
