# Supplementary material for: CRISPR/Cas9-mediated mutation of OsSWEET14 in rice cv. Zhonghua11 confers resistance to Xanthomonas oryzae pv. oryzae without yield penalty
Source: BMC Plant Biol. 2020 Jul 3;20:313. doi: 10.1186/s12870-020-02524-y (PMC7333420; doi:10.1186/s12870-020-02524-y)
Supplement: Supplementary file 6 — Additional file 6. Agronomic traits of CR-S14. [file 12870_2020_2524_MOESM6_ESM.pdf]

Additional file 6 Agronomic traits of *CR-S14*.

	**ZH11**[6]	***CR-S14-2***	***CR-S14-6***	***CR-S14-9-I***	***CR-S14-9-II***
Stem diameter[1]	4.170±0.46 (*n*=138)	4.091±0.45 (*n*=129, *P*=0.1585)	4.062±0.43 (*n*=94, *P*=0.0732)	4.097±0.47 (*n*=70, *P*=0.2881)	4.090±0.43 (*n*=109, *P*=0.1644)
Plant height[2]	88.08±5.63 (*n*=72)	95.03±5.43 (*n*=35, *P*=2.21×10^{-8})	88.40±7.19 (*n*=26, *P*=0.8182)	95.74±4.32 (n=17, *P*=1.11×10^{-6})	95.14±5.69 (*n*=28, *P*=1.880^{-7})
1,000-grain weight[3]	21.66±2.02 (*n*=49)	22.65±2.37 (*n*=38, *P*=0.0392)	21.78±2.37 (*n*=23, *P*=0.8312)	22.20±2.133 (*n*=17, *P*=0.3530)	22.80±2.36 (*n*=27, *P*=0.0303)
Seed setting rate[4]	81.18±8.78 (*n*=49)	84.63±8.356 (*n*=35, *P*=0.0744)	80.89±9.54 (*n*=21, *P*=0.9012)	80.6±9.18 (*n*=16, *P*=0.8192)	80.56±10.77 (*n*=27, *P*=0.7845)
Yield per main panicle[5]	2.103±0.36 (*n*=49)	2.112±0.50 (*n*=37, *P*=0.9251)	2.143±0.57 (*n*=58, *P*=0.6733)	2.159±0.32 (*n*=16, *P*=0.5832)	2.058±0.52 (*n*=27, *P*=0.6542)

[1]The upper diameter of the second stem segment of every productive tiller was measured as the stem diameter. At least 15 plants were assessed. *n*, the total number of productive tillers that have been measured. *P*, *P*-value calculated by two-tailed Student's *t* test against Zhonghua 11.

[2]The length of the mature rice plant from ground to the tip of the panicle was measured as the plant height. *n*, the total number of plants measured. *P*, *P*-value calculated by two-tailed Student's *t* test against Zhonghua 11.

[3]The 1,000-grain weight of main panicle was weighed. *n*, the total number of plants assessed. *P*, *P*-value calculated by two-tailed Student's *t* test against Zhonghua 11.

[4]The seed setting rate of main panicle was measured. *n*, the total number of plants assessed. *P*, *P*-value calculated by two-tailed Student's *t* test against Zhonghua 11.

[5]The weight of filled grain of main panicle was weighed as the yield per main panicle. *n*, the total number of plants assessed. *P*, *P*-value calculated by two-tailed Student's *t* test against Zhonghua 11.

[6]ZH11, Zhonghua 11;
