# Supplementary material for: CRISPR/Cas9-mediated mutation of OsSWEET14 in rice cv. Zhonghua11 confers resistance to Xanthomonas oryzae pv. oryzae without yield penalty
Source: BMC Plant Biol. 2020 Jul 3;20:313. doi: 10.1186/s12870-020-02524-y (PMC7333420; doi:10.1186/s12870-020-02524-y)
Supplement: Supplementary file 7 — Additional file 7. Source of Xoo strains. [file 12870_2020_2524_MOESM7_ESM.pdf]

Additional file 7 Source of *Xoo* strains.

**Strain**	**Country**	**Source**
GD1358	China	provided by Dr. Zhongchao Yin (Temasek Life Sciences Laboratory)
JS4906	China	provided by Dr. Zhongchao Yin (Temasek Life Sciences Laboratory)
HB17	China	provided by Dr. Zhongchao Yin (Temasek Life Sciences Laboratory)
HB21	China	provided by Dr. Zhongchao Yin (Temasek Life Sciences Laboratory)
HLJ72	China	provided by Dr. Zhongchao Yin (Temasek Life Sciences Laboratory)
NX42	China	provided by Dr. Zhongchao Yin (Temasek Life Sciences Laboratory)
HN1-2	China	isolated from rice fields in Guangdong Province by our lab
LC-4	China	isolated from rice fields in Guangdong Province by our lab
IV-1	China	isolated from rice fields in Guangdong Province by our lab
PXO79	Philippines	provided by Dr. Zhongchao Yin (Temasek Life Sciences Laboratory)
PXO86	Philippines	provided by Dr. Zhongchao Yin (Temasek Life Sciences Laboratory)
PXO71	Philippines	provided by Dr. Zhongchao Yin (Temasek Life Sciences Laboratory)
Aust2031	Australia	provided by Dr. Zhongchao Yin (Temasek Life Sciences Laboratory)
T7174	Japan	provided by Dr. Zhongchao Yin (Temasek Life Sciences Laboratory)
A3857	India	provided by Dr. Zhongchao Yin (Temasek Life Sciences Laboratory)
A3842	India	provided by Dr. Zhongchao Yin (Temasek Life Sciences Laboratory)
