# Supplementary material for: CRISPR/Cas9-mediated mutation of OsSWEET14 in rice cv. Zhonghua11 confers resistance to Xanthomonas oryzae pv. oryzae without yield penalty
Source: BMC Plant Biol. 2020 Jul 3;20:313. doi: 10.1186/s12870-020-02524-y (PMC7333420; doi:10.1186/s12870-020-02524-y)
Supplement: Supplementary file 8 — Additional file 8. Primers used in this study. [file 12870_2020_2524_MOESM8_ESM.pdf]

Additional file 8 Primers used in this study.

Primer	Sequence (5'-3')	remarks
OsU3-S14E1	GGGATGCTGAAGAGACATGTGC CACGGATCATCTGC	Primers for *OsSWEET14*-CRISPR-Cas9 construction
U6b-S14E3	ACTGGAACCCCTGCGTCGACCA ACACAAGCGGCAGC	
gR-S14E1	CATGTCTCTTCAGCATCCCGTTT TAGAGCTAGAAAT	
gR-S14E3	GTCGACGCAGGGGTTCCAGTGTT TTAGAGCTAGAAAT	
CR-S14-qRT-F	TTCTTGGTTGGGTCTGCGTT	qRT-PCR primers
CR-S14-qRT-R	GAGGGAGAAGGAGAGCGAGA	
UBQ5-F	AACCACTTCGACCGCCACT	
UBQ5-R	GTTCGATTTCCTCCTCCTTCC	
Target-TalC-F	CCCATGCATTGAGGACAGAGT	Primers for mutant allele amplification and sequencing
Target-TalC-R	TGGCAACAAAAGTGGAACAT	
Target-S14E3-F	TTATAACGTCCGTCGCATTTT	
Target-S14E3-R	TTGGGGGCGTAGACGAGGTAGA	
S14-P-F	AACGTCGACAGGGATGAAATAC CAAAACACA	For *pOsSWEET14:GUS* construction
S14-P-R	ACCAGATCTATGATCAGGCAAT AAAGCAGAG	
